# Characteristics of patients admitted to a Psychiatric Home Hospitalization Unit and burden felt by caregivers

**DOI:** 10.1192/j.eurpsy.2023.1909

**Published:** 2023-07-19

**Authors:** J. Marti-Bonany, O. Garcia, D. Tolosa, R. Romar, G. Mateu, D. Garcia, R. Sanchez, M. G. Hurtado, M. Campillo, C. Monserrat, M. Roldan

**Affiliations:** INAD-Parc de Salut Mar, Santa Coloma de Gramenet, Spain

## Abstract

**Introduction:**

Severe mental illnesses characterized by periods of relapse that require intensive resource management. Caregivers of schizophrenia and bipolar disorder patients feel a considerable burden of care (Tanna *et al*. Ind Psychiatry J 2021, 30(2):299-304).

The main objective of Hospital at home for psychiatric patients is to provide intense care to patients with severe mental disorders at home as an alternative to acute admission (Alba et al. Rev Psiquiatr Salud Ment. 2019, 12 (4) 207-212).

**Objectives:**

The aim of this study is to describe the characteristics of patients attended at the Psychiatric Home Hospitalization Unit of our hospital (HAD-CAEM) and to assess the of burden of care that caregivers feel while giveing care to this patients.

**Methods:**

Data were collected retrospectively at admission and discharge of all patients treated at HAD-CAEM between August 2018 to March 2022. Incomes of patients who met DSM-5 criteria for schizophrenia, bipolar disorder and major depressive disorder. Severity of disease and patient’s level of functionality was evaluated with the global assessment of functioning scale (GAF) and the Clinical Global Impression Scale (CGI). Burden Caregivers was evaluated with The Zarit Caregiver Burden Scale (ZCBS). Statistical analysis was performed by using SPSS program.

**Results:**

109 patients were included in the study. 49.5% were women. The mean age was 48 years (SD 18.47 years). 44% met criteria for schizophrenia, 25.7% for depressive disorder, and 30.3% for bipolar disorder. Most of them lived with their own family (47.7%); had secondary education (51.4%) and were unemployed (33%). 81% had a history of at least one admission to an acute psychiatric unit.

The mean duration of admission in HAD-CAEM was 33.8 days (SD 15.72 days), with a mean follow-up of 8.75 visits (SD 3.58 visits).

The mean CGI severity item at admission was 4.36 and there was an improvement at the time of discharge according to the CGI improvement item (mean CGI-I=2.43).

The GAF scale on admission was 46.74 (SD 11.2) and on discharge 64.24 (SD 13.85), showing an improvement of 17.5 points at discharge (p<0.001).

The mean ZCBS of the sample was 48.21 (SD 15.11). Mean ZCBS in Schizophrenia group (n=22) was 46.13 (SD 16.53), in depressive group (n=18) was 43.61 (SD 12.89) and for bipolar group (n=17) was 55.76 (SD 13.19). A statistical test is performed with ANOVA, showing significant differences between groups (p=0.039). Post-hoc analyzes show significant differences between bipolar disorder group and the depressive disorder group (p=0.04). No significant differences are found between the other groups.

**Conclusions:**

Caregivers of schizophrenia, depressive and bipolar disorder patients feel a considerable burden of care. ZCBS was administered to the caregivers on the last day of admission, when the patient presented clinical and functional improvement. More studies are needed to support these results.

**Disclosure of Interest:**

None Declared

